# The Broad Anti-AML Activity of the CD33/CD3 BiTE Antibody Construct, AMG 330, Is Impacted by Disease Stage and Risk

**DOI:** 10.1371/journal.pone.0135945

**Published:** 2015-08-25

**Authors:** Kimberly H. Harrington, Chelsea J. Gudgeon, George S. Laszlo, Kathryn J. Newhall, Angus M. Sinclair, Stanley R. Frankel, Roman Kischel, Guang Chen, Roland B. Walter

**Affiliations:** 1 Clinical Research Division, Fred Hutchinson Cancer Research Center, Seattle, Washington, United States of America; 2 Amgen, Inc., Seattle, Washington, United States of America; 3 Amgen, Inc., Thousand Oaks, California, United States of America; 4 Amgen, Inc., Rockville, Maryland, United States of America; 5 Amgen Research (Munich) GmbH, Munich, Germany; 6 Department of Medicine, Division of Hematology, University of Washington, Seattle, Washington, United States of America; 7 Department of Epidemiology, University of Washington, Seattle, Washington, United States of America; Queen's University Belfast, UNITED KINGDOM

## Abstract

The CD33/CD3-bispecific T-cell engaging (BiTE) antibody construct, AMG 330, potently lyses CD33+ leukemic cells *in vitro*. Using specimens from 41 patients with acute myeloid leukemia (AML), we studied the factors that might contribute to clinical response or resistance. For this purpose, thawed aliquots of primary AML samples were immunophenotypically characterized and subjected to various doses of AMG 330 in the presence or absence of healthy donor T-cells. After 48 hours, drug-specific cytotoxicity was quantified and correlated with CD33 expression levels, amounts of T-cells present, and other disease characteristics. AMG 330 caused modest cytotoxicity that was correlated with the amount of autologous T-cells (*P* = 0.0001) but not CD33 expression, as AMG 330 exerted marked cytotoxic effects in several specimens with minimal CD33 expression. With healthy donor T-cells added, AMG 330 cytotoxicity depended on the drug dose and effector:target (E:T) cell ratio. High cytotoxic activity was observed even with minimal CD33 expression, and AMG 330 cytotoxicity and CD33 expression correlated only at high E:T cell ratio and high AMG 330 doses (*P*<0.003). AMG 330 resulted in significantly higher cytotoxicity in specimens from patients with newly diagnosed AML than those with relapsed/refractory disease despite similar levels of CD33 on myeloblasts. AMG 330 cytotoxicity also appeared greater in specimens from patients with favorable-risk disease as compared to other specimens. Together, our data demonstrate that AMG 330 is highly active in primary AML specimens across the entire disease spectrum, while suggesting the presence of yet undefined, CD33-independent, relative resistance mechanisms in specific patient subsets.

## Introduction

Over the last several decades, the outcomes for many patients with acute myeloid leukemia (AML) have significantly improved. However, much of this progress is due to advances in supportive care, which have rendered curative-intent chemotherapy and allogeneic hematopoietic cell transplantation (HCT) safer and paved the way for intensified treatment algorithms [1–3]. Despite these improvements, a large proportion of AML patients is currently still expected to die of their disease or treatment-related complications. The need for effective yet well-tolerated new therapies for this disease is therefore unquestioned.

Antibodies raised high expectations as a potent means of eliminating malignant cells with limited non-specific toxicities. Because of well-defined surface antigens and easy tumor cell accessibility, AML has been a paradigm for the therapeutic use of unconjugated and drug/toxin-loaded antibodies. Hitherto most exploited is CD33, a myeloid differentiation antigen displayed on at least a subset of AML blasts in most patients and, possibly, on leukemia stem cells in some [4, 5]. Indeed, among the few novel drugs have shown a benefit in randomized trials is the CD33 antibody-drug conjugate, gemtuzumab ozogamicin (GO), which has shown to improve survival in subsets of patients when added to intensive induction chemotherapy [6, 7]. Nevertheless, CD33 is a challenging target for toxin-loaded antibodies due to its relatively low abundance on the cell surface and slow internalization. Together with drug transporter activity in AML cells, such limitations may explain why GO is ineffective in many patients and is currently no longer commercially available in most countries [4, 5].

Many of the shortcomings of antibody-drug conjugates such as GO may not pertain to bispecific T-cell engaging (BiTE) antibody constructs. This relatively novel subclass of therapeutics combines the minimal binding domains of an anti-CD3 antibody and an antibody directed against a surface antigen present on tumor cells fused in the form of a tandem single-chain Fv antibody on a single stable polypeptide chain of approximately 55 kDa [8–13]. A first candidate built on this platform to target AML is the CD33/CD3-directed molecule, AMG 330. Several recent preclinical studies have demonstrated that AMG 330 is highly potent in causing cytolysis of CD33^+^ AML cell lines or primary human AML cells in the presence of healthy donor T-cells or autologous T-cells from AML patients at low effector-to-target (E:T) cell ratio [14–17]. In experiments with well-defined AML cell lines and genetically engineered sublines, we identified target antigen density, antibody dose, and E:T cell ratio but not drug transporter activity as critical determinants for the activity of AMG 330 in cell line models of AML [16]. So far, however, systematic preclinical investigations in larger sets of specimens from patients with AML that could identify response biomarkers and inform the design of clinical investigations testing AMG 330 have not yet been performed. With this aim in mind, we used a set of nearly 50 primary AML specimens to determine the disease characteristics that impact the activity of AMG 330 against primary AML cells *in vitro*.

## Materials and Methods

### Primary human AML cells

Frozen aliquots of Ficoll-isolated mononuclear cells from pretreatment (“diagnostic”) peripheral blood or bone marrow specimens from adult patients with AML were obtained from repositories at Fred Hutchinson Cancer Research Center (FHCRC). We used the 2008 WHO criteria to define AML [18] and the refined United Kingdom Medical Research Council (MRC) criteria to assign cytogenetic risk [19]. Patients provided written informed consent for the collection and use of their biospecimens for research purposes under protocols approved by the FHCRC Institutional Review Board. Clinical data were de-identified in compliance with Health Insurance Portability and Accountability Act regulations.

### Healthy donor T-cells

Unstimulated mononuclear cells were collected via leukapheresis from a single healthy adult volunteer who provided written informed consent for the collection and use of these cells for research purposes under protocols approved by the Western Institutional Review Board (Olympia, WA). T-cells were enriched through magnetic cell sorting (Pan T-Cell Isolation Kit II; Miltenyi Biotec, Auburn, CA), and then frozen in aliquots and stored in liquid nitrogen. For use, cells were labeled with 3 μM CellVue Burgundy (eBioscience, San Diego, CA) [16].

### Immunophenotypic characterization of primary AML specimens

Cells were stained with directly labeled antibodies recognizing CD33 (clone P67.6), CD3 (clone SK7), CD34 (clone 8G12), and CD38 (clone HB7; all from BD Biosciences, San Jose, CA) and CD45 (clone HI30; eBioscience). The activities of P-glycoprotein (Pgp), multidrug resistance protein (MRP), and breast cancer resistance protein (BCRP) were determined using a commercial flow cytometric kit (eFLUXX-ID, ENZO Life Sciences, Plymouth Meeting, PA). To identify nonviable cells, samples were stained with 4',6-diamidino-2-phenylindole (DAPI). At least 10,000 events were acquired on a Canto II flow cytometer (BD Biosciences), and DAPI^-^ cells analyzed using FlowJo (Tree Star, Ashland, OR).

### Quantification of drug-induced cytotoxicity on AML blasts

Cells were incubated at 37°C (in 5% CO_2_ and air) in 96-well round bottom plates (BD Falcon) at 10,000 cells/well in 225 μL Iscoves’ Modified Dulbecco’s medium (IMDM) supplemented with 20% fetal bovine serum (FBS), cytokines (10 ng/mL each of IL-3, SCF, G-CSF, and GM-CSF; all from Life Technologies, Grand Island, NY), and containing various concentrations of AMG 330 (provided by Amgen, Amgen Research Munich GmbH, Munich, Germany) as well as T-cells at different E:T cell ratios. In previous studies with human AML cell lines and engineered sublines, we used E:T cell ratios as high as 5:1 and 10:1 [16]. However, we found these higher E:T cell ratios to be less informative than lower E:T cell ratios for the characterization of inter-sample sensitivities to BiTE-induced cytotoxicity. Therefore, and because of limited number of blasts available from each patient, we restricted our analyses to E:T cell ratios not exceeding 3:1 in the current analyses. After 48 hours, cell numbers and drug-induced cytotoxicity, using DAPI to detect non-viable cells, were determined using a LSRII cytometer (BD Biosciences) and analyzed with FlowJo. AML cells were identified by forward/side scatter properties and, in experiments where healthy donor T-cells were added, negativity for CellVue Burgundy dye (see S1 Fig) [16].

### Long-term in vitro culture and colony-forming cell (CFC) assays of AMG 330-treated primary AML specimens

To test the effect of AMG 330 on immature AML cell populations, unsorted cells from thawed AML specimens were cultured in IMDM supplemented with 20% FBS and 10 ng/mL each of IL-3, SCF, G-CSF, and GM-CSF containing no AMG 330 or AMG 330 at either 100 pg/mL or 500 pg/mL (because of limited cell numbers available, experiments could only be conducted with 2 different concentrations of AMG 330) together with CellVue Burgundy dye-labeled T-cells at an E:T = 3:1 cell ratio. After 48 hours, CellVue Burgundy dye-negative primary AML cells were separated using a FACSAria flow cytometer (BD Biosciences), and cells were cultured in IMDM with 20% FBS containing the recombinant human cytokines SCF, interleukin-6, FLT3 ligand, and thrombopoietin (all from Invitrogen), StemRegenin-1 (SR1, Cellagen Technology, San Diego, CA), and penicillin-streptomycin [20]. Cultures were replenished with fresh medium weekly and kept at 37°C in 3% O_2_ and 5% CO_2_ for 4 weeks. Defined fractions of the cultures were removed after 2 and 4 weeks and subjected to CFC assays. After 10–14 days at 37°C in 3% O_2_ and 5% CO_2_, colony forming units-granulocyte and/or monocyte (CFU-GM) of at least 30–50 cells were quantified [20].

### Statistical considerations

Linear median fluorescence intensity (MFI) values were used to quantify CD33 expression levels. Drug-induced specific cytotoxicity is presented as: % cytotoxicity = 100 x (1 –live target cells_treated_/live target cells_control_) [16]. Drug transporter activity was calculated as multidrug resistance activity factor (MAF) per manufacturer’s recommendations as: MAF = 100 × (fluorescence intensity_drug inhibitor_—fluorescence intensity_DMSO/control_)/ fluorescence intensity_drug inhibitor_, with values <20 considered to indicate multidrug resistance negative, and values > considered to indicate multidrug resistance positive. Results from cytotoxicity assays are presented as mean values ± standard error of the mean (SEM). Spearman nonparametric correlation was used to estimate correlations between continuous sample characteristics and drug-induced specific cytotoxicity. The dependency of cytotoxicity on the drug dose (at E:T = 1:3) or on the E:T cell ratio (at dose = 500 pg/mL) was evaluated using a repeated measure ANOVA model. In addition to BiTE antibody construct dose or E:T cell ratio, the model also included gender, age, CD33 expression, and autologous T-cells to account for potential effects. Repeated measure ANOVA method was also employed to assess the difference in AMG 330-induced cytotoxicity between disease stage and cytogenetic risk groups. The analysis was performed at E:T cell ratios of 1:1 and 1:3 separately. The ANOVA model included factors of AMG 330 dose, disease stage, and cytogenetic risk, as well as gender, age, CD33 expression, and autologous T-cells as covariates. All *P*-values are two-sided. Statistical analyses were performed using Prism 6.0e (GraphPad; La Jolla, CA) or SAS 9.3 (SAS Institute Inc., Cary, NC).

## Results

### Selection and characteristics of primary AML specimens

We obtained specimens from 49 AML patients for our studies. Upon thaw, 47 had >40% AML blasts, as determined by flow cytometry based on CD45/side-scatter properties. Forty-one of these 47 specimens had >50% viable cells upon thaw and >30% viable cells after 48 hours in cytokine-containing liquid culture (S2 Fig) and were included in our analyses. Median age of the patients was 65.3 (range: 23.9–80.0) years; cytogenetic disease risk was favorable in 2, intermediate in 29, and adverse in 10. Information on the mutation status of NPM1, FLT3, and CEBPA was incomplete; however, 1 sample was known to be *CEBPA* ^double-mutant^, and 2 samples were *NPM1* ^pos^/*FLT3-ITD* ^neg^, and were considered as favorable-risk AML specimens in subset analyses. The median percentage of myeloid blasts and CD3+ T-cells in the studied specimens was 87.1% (range: 55.1–97.0%) and 2.0% (range: 0–27.3%), respectively, and the median sample viability after 48 hours in culture was 76.7% (range: 31.1–93.5%). Twenty-one of the patients had newly diagnosed AML, whereas 20 either had relapsed (n = 11) or refractory (n = 9) disease at the time of specimen collection; as summarized in Table 1, basic characteristics of the specimens from patients with newly diagnosed AML were similar to those with relapsed/refractory disease with regard to CD33 expression on myeloid blasts, amount of autologous T-cells, proportion of myeloid blasts, and culture viability. These characteristics were also relatively similar when specimens were grouped by cytogenetic/molecular risk except that the CD33 expression on myeloid blasts was lower in adverse-risk specimens (S1 Table).

**Table 1:** Patient Characteristics.

	All patients	Newly diagnosed AML	Relapsed/refractory AML
	(n = 41)	(n = 21)	(n = 20)
**Median age (range), years**	65.3 (23.9–80.0)	65.7 (23.9–80.0)	62.9 (26.2–76.4)
**Cytogenetic/molecular risk**			
Favorable	2	2	--
Intermediate	29	14	15
*CEBPA* ^*double-mutant*^	1	1	--
*NPM1* ^*pos*^ */FLT3-ITD* ^*neg*^	2	1	1
Adverse	10	5	5
**Specimen source**			
Bone marrow	20	9	11
Peripheral blood	21	12	9
**Median % blasts (range)**	87.1 (55.1–97.0)	87.9 (58.7–95.5)	86.4 (55.1–97.0)
**Median CD33 expression on blasts (range)**	849 (7–5,356)	837 (30–5,356)	1,018 (7–2,567)
**Median % T-cells (range)**	2.0 (0–27.3)	2.8 (0–11.9)	2.0 (0.2–27.3)
**Median Pgp activity on blasts (range)**	36.2 (0–66.5)	29.5 (0–64.6)	37.9 (0–66.5)
**Median % viability at 48 hours (range)**	76.7 (31.1–93.5)	71.8 (31.1–93.2)	80.4 (32.5–93.5)

### AMG 330 engages autologous T-cells to lyse AML cells

**Fig 1:**
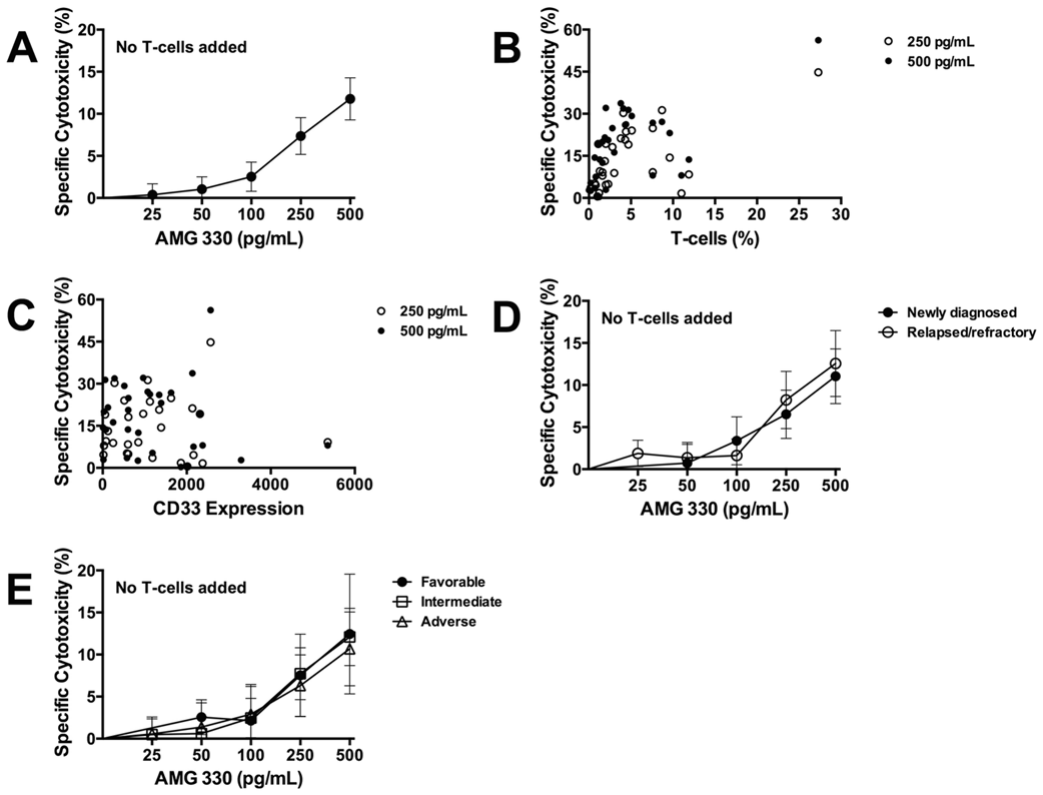
AMG 330-induced cytotoxicity without addition of healthy donor T-cells. **(A)** 41 primary AML specimens were incubated with increasing concentrations of AMG 330. After 48 hours, cell counts were determined and cytotoxicity was assessed with DAPI staining to quantify drug-specific cytotoxicity. Results are shown as mean±SEM. **(B)** Relationship between percentage of autologous T-cells and AMG 330-induced cytotoxicity. **(C)** Relationship between CD33 expression on leukemic blasts (expressed as arbitrary fluorescence intensity) and AMG 330-induced cytotoxicity. **(D)** AMG 330-induced cytotoxicity, stratified by disease stage (newly diagnosed AML [n = 21] and relapsed/refractory AML [n = 20]). Results are shown as mean±SEM. **(E)** AMG 330-induced cytotoxicity, stratified by disease risk (favorable-risk [n = 5]; intermediate-risk [n = 26]); and adverse-risk [n = 10]). Results are shown as mean±SEM.

The addition of AMG 330 to AML specimen cultures resulted in dose-dependent cytotoxicity (e.g. specific cytotoxicity after 48 hours with AMG 330 at 500 pg/mL: 11.8±2.5%; Fig 1A), demonstrating that autologous T-cells contained in the specimens from patients with active AML can be engaged to lyse leukemic cells. As depicted in Fig 1B, AMG 330-induced cytotoxicity was statistically highly significantly correlated with the amount of autologous T-cells (at 250 pg/mL: Spearman rank correlation coefficient r = 0.595 [95% confidence interval: 0.343–0.767], *P*<0.0001; at 500 pg/mL: r = 0.564 [95% confidence interval: 0.301–0.747], *P* = 0.0001). AMG 330 exerted marked cytotoxic activity in several specimens with very low CD33 expression levels on AML blasts, and we found no significant correlation between CD33 expression levels and drug-induced cytotoxicity (at 250 pg/mL: r = 0.012 [-0.306–0.327], *P* = 0.94; at 500 pg/mL: r = -0.048 [-0.359–0.272], *P* = 0.77; Fig 1C). In analyses of patient subsets, AMG 330 resulted in similar cytotoxicity in specimens from patients with newly diagnosed AML and those with relapsed/refractory disease (Fig 1D). Likewise, we observed no statistically significant differences between the cytotoxic effects of AMG 330 in favorable-risk, intermediate-risk, and adverse-risk AML specimens (Fig 1E).

### Activity of AMG 330 in the presence of added healthy donor T-cells

**Fig 2:**
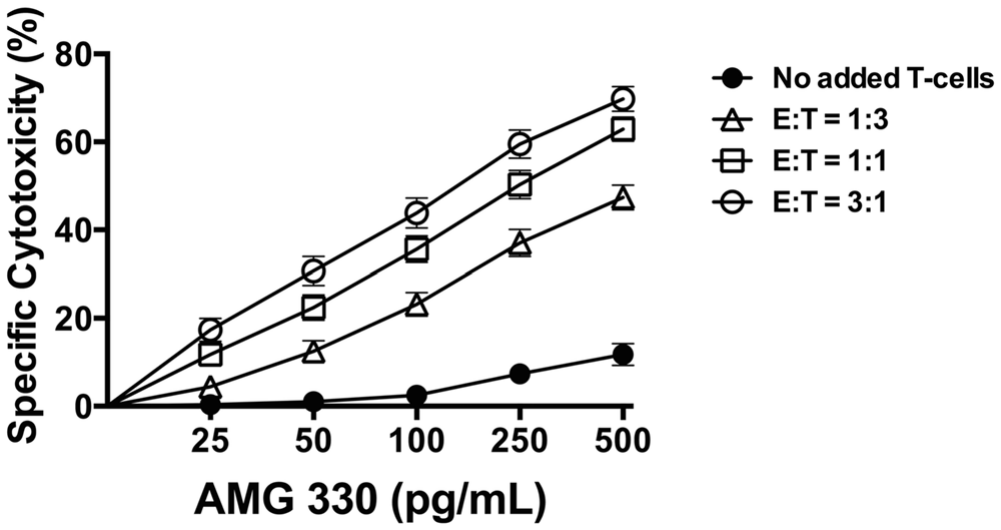
AMG 330-induced cytotoxicity in the presence of healthy donor T-cells. Forty-one primary AML specimens were incubated with increasing concentrations of AMG 330 and T-cells from a single healthy donor at various effector:target (E:T) cell ratios as indicated. After 48 hours, cell counts were determined and cytotoxicity was assessed with DAPI staining to quantify drug-specific cytotoxicity. Results are shown as mean±SEM.

**Fig 3:**
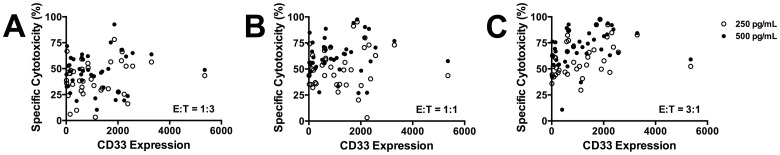
Relationship between CD33 expression and AMG 330-induced cytotoxicity. Relationship between CD33 expression on leukemic blasts (expressed as arbitrary fluorescence intensity) and drug-induced cytotoxicity with AMG 330 at 250 pg/mL (open symbol) and 500 pg/mL (closed symbol) in the presence of T-cells from a single healthy donor at an E:T cell ratio of **(A)** 1:3, **(B)** 1:1, and **(C)** 3:1, determined after 48 hours.

As shown in Fig 2, the cytotoxic activity of AMG 330 was strictly dependent on the drug dose (e.g. *P*<0.0001 at E:T = 1:3) and the E:T cell ratio (e.g. *P*<0.0001 at 500 pg/mL). However, high activity of AMG 330 was even observed in specimens with very low CD33 expression on AML blasts. Similar to the experiments in which no healthy donor T-cells were added, there was no significant correlation between drug-induced cytotoxicity and CD33 expression levels on AML blasts at lower E:T cell ratios (with E:T = 1:3, *P* = 0.86 and *P* = 0.50 at 250 and 500 pg/mL, respectively; with E:T = 1:1, *P* = 0.43 and *P* = 0.16 at 250 and 500 pg/mL, respectively; Fig 3A and 3B). On the other hand, in experiments in which an E:T cell ratio of 3:1 was used, there was a statistically significant correlation between CD33 expression on AML blasts and AMG 330-induced cytotoxicity (at 250 pg/mL: r = 0.457 [0.165–0.676], *P* = 0.0027; at 500 pg/mL: r = -0.465 [0.174–0.681], *P* = 0.0022; Fig 3C).

**Fig 4:**
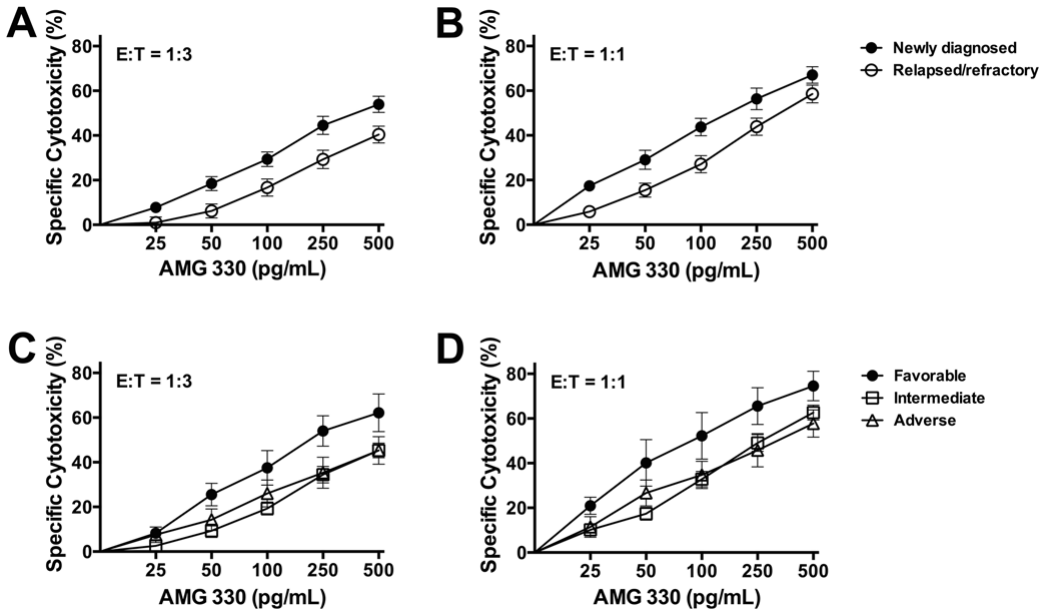
AMG 330-induced cytotoxicity in the presence of healthy donor T-cells, stratified by disease stage and risk. AMG 330-induced cytotoxicity at 48 hours, stratified by **(A, B)** disease stage (newly diagnosed AML [n = 21] and relapsed/refractory AML [n = 20]), and **(C, D)** cytogenetic/molecular disease risk (favorable-risk [n = 5]; intermediate-risk [n = 26]); and adverse-risk [n = 10]) in the presence of T-cells from a single healthy donor at an E:T cell ratio of 1:3 and 1:1, as indicated.

In analyses of patient subsets, AMG 330, in the presence of healthy donor T-cells, resulted in significantly higher cytotoxicity in specimens from patients with newly diagnosed AML (n = 21) than those with relapsed/refractory disease (n = 20; *P* = 0.022 at E:T = 1:3 and *P* = 0.045 at E:T = 1:1; Fig 4A and 4B). Furthermore, AMG 330-induced cytotoxicity was higher in specimens from patients with favorable-risk disease as compared to those with intermediate-or adverse risk disease (Fig 4C and 4D). There was, however, no evidence that the activity of AMG 330 was directly related to the patient age; in fact, in some of the experimental conditions, there was a positive correlation between AMG 330 induced cytotoxicity and age of the patient whose specimen was studied (with E:T = 1:3, *P* = 0.04 at 250 pg/mL; with E:T = 1:1, *P* = 0.03 at 250 pg/mL; S3 Fig). Likewise, there was no evidence that the activity of AMG 330 was lower in specimens with higher Pgp activity (all *P*>0.13; S4 Fig).

### Activity of AMG 330 in the subset of AML specimens with the highest in vitro baseline viability

The results presented thus far were obtained in the set of 41 primary AML specimens that we selected based on minimum requirements on viability both at the time of thaw (i.e. >50% viable cells) as well as after 48 hours in cytokine-containing liquid culture (i.e. >30% viable cells). As these (arbitrarily defined) requirements could be criticized as not being stringent enough, we performed additional analyses in the subset of 25 primary AML specimens that showed a viability of >70% both at thaw as well as after 48 hours in cytokine-containing liquid culture (S2 Fig and S2 table). As summarized in S5 and S6 Figs, findings on this sample subset were congruent with those obtained in the larger sample set. Specifically, in the absence of added healthy donor T-cells, a dose-dependent cytotoxic effect of AMG 330 was observed that was dependent on the amount of autologous T-cells but not expression levels of CD33 on myeloid blasts. Addition of healthy donor T-cells at increasing E:T cell ratios step-wise increased AMG 330-induced cytotoxicity. Despite similar CD33 expression levels between specimens from patients with newly diagnosed AML (n = 12) and those with relapsed/refractory disease (n = 13), the cytotoxic effects with AMG 330 were greater in specimens derived from patients with newly diagnosed AML.

### Effect of AMG 330 on colony-forming cells (CFCs)

**Fig 5:**
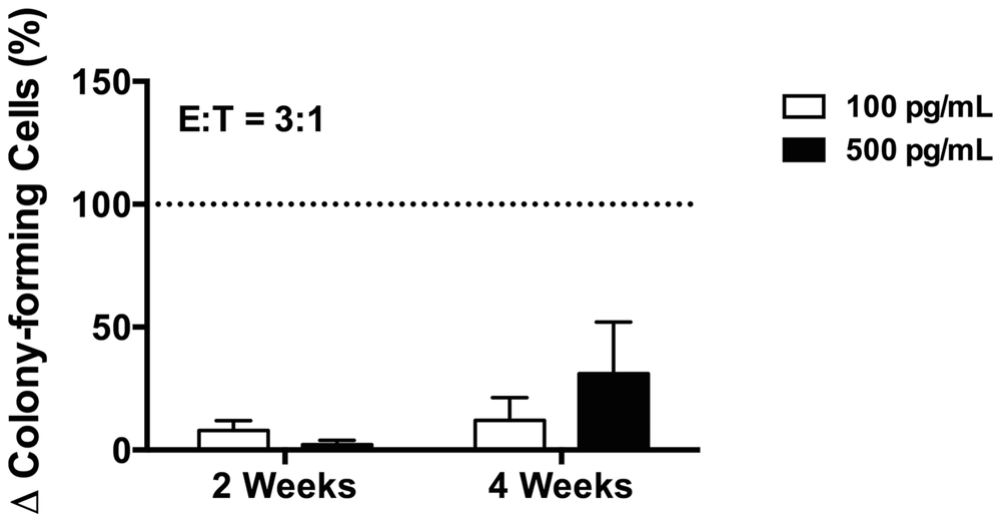
Effect of AMG 330 on colony-forming cells (CFC). AML cells were incubated without AMG 330 or in the presence of 100 pg/mL or 500 pg/mL of AMG 330 together with healthy donor T-cells (labeled with CellVue Burgundy dye) at an E:T cell ratio of 3:1. After 48 hours, CellVue Burgundy dye-negative primary AML cells were separated by FACS and subsequently cultured for up to 4 weeks. After 2 and 4 weeks, aliquots of cultures cells were subjected to CFC assays. 10–14 days later, CFU-GMs were quantified and plating efficiency determined. Data (CFU-GMs/10,000 plated cells) from aliquots incubated with AMG 330 and healthy donor T-cells are expressed relative to aliquots incubated without AMG 330 (with no-AMG 330 controls set at 100%). Depicted are data from 16 AML specimens that yielded CFU-GM growth without AMG 330.

Finally, to test the effect of AMG 330 on immature AML cell populations, we incubated unsorted cells from 28 AML specimens with or without AMG 330 in the presence of healthy donor T-cells. After 48 hours, healthy donor T-cells were removed by FACS, and remaining patient-derived cells placed in liquid culture for 4 weeks. After 2 and 4 weeks, aliquots of cultured cells were then subjected to CFC assays, and CFU-GMs quantified after 10–14 days. A total of 16 specimens each yielded CFU-GM growth after 2 and 4 weeks, respectively, of *in vitro* culture in the absence of AMG 330 treatment. As shown in Fig 5, AMG 330 either at 100 pg/mL or 500 pg/mL significantly reduced the amount of CFU-GMs after *in vitro* culture relative to aliquots that were not treated with AMG 330.

## Discussion

Bispecific constructs that harness the immune system in the elimination of cancer cells are a long-pursued strategy to improve the efficacy of anti-tumor antibodies. Many bispecific construct modalities have been explored over the years, but their success was limited by suboptimal effector cell recruitment and challenges with large-scale, clinical-grade antibody production [11, 21]. Undoubtedly, interest in this therapeutic approach has been renewed with the demonstration that very low doses of the CD19/CD3 BiTE antibody construct, blinatumomab, can eliminate target cells in patients with non-Hodgkin’s lymphomas [22]. Clinical studies with blinatumomab, showing a high response and relapse-free survival rate among adults with CD19^+^ acute lymphoblastic leukemia (ALL) that persisted or relapsed after chemotherapy [23–25], suggest the potential of these molecules for acute leukemias.

With the expression of CD33 on myeloid blasts in most AML cases, and the survival improvement seen with GO in some patient subsets with this disease, AMG 330 is a logical first BiTE antibody construct for the treatment of human AML [5, 13]. Recent preclinical studies from other groups and ours have demonstrated that AMG 330 effectively redirects T-cells to destroy CD33^+^ AML cells [14–17]; of note, unlike bivalent antibodies, continuous exposure to AMG 330 at cytotoxic concentrations does not lead to down-modulation of CD33 expression on AML cells [16]. While these initial investigations have included studies on primary AML cells, detailed explorations of the factors that might contribute to clinical response or resistance have not been conducted.

The goal of the current studies was to investigate the characteristics that affect the activity of AMG 330 against primary AML cells *in vitro*. As we were interested in discerning differences between AML cells from individual patients, we studied the effects of AMG 330 after a short incubation period (48 hours, consistent with our previous studies in AML cell lines [16]) and at low E:T cell ratios (up to E:T = 3:1) and relatively limited antibody concentrations (up to 500 pg/mL). To facilitate comparisons, we also used exogenous T-cells from a single healthy donor for all experiments performed. Using a large set of primary patient specimens, our studies demonstrate that AMG 330 is highly active against human AML blasts and less mature cell subsets such as CFCs at extremely low concentrations (≤500 pg/mL). Drug-specific cytotoxicity was observed in the presence of residual autologous T-cells and by the addition of controlled amounts of healthy donor T-cells. Since we selected specimens with >40% myeloid blasts for our studies and we did not supplement specimens with additional aliquots of patient-derived lymphocytes, the calculated E:T cell ratios in the experiments that we conducted with autologous T-cells alone were very low. Specifically, across all 41 studied specimens, the median autologous T-cell-to-AML cell ratio was 1:43 with a range of 1:2 to as low as 1:>2,700. Although we did not enumerate T-cells after the 48-hour assay period in the current studies, our previous studies have failed to show significant expansion of T-cell populations over this short culture time [16]. Thus, our data indicate that AMG 330 can efficiently engage autologous T-cells. When studying AMG 330 either with autologous T-cells alone or with added healthy donor T-cells, drug-induced cytotoxicity was correlated to both the drug concentration used as well as the amount of T-cells present, confirming the AMG 330 dose and E:T cell ratio as two critical determinants of the anti-leukemic activity of this BiTE antibody construct. In contrast, but similar to our previous studies with AML cell lines [16], our current studies indicate that drug transporter activity, in particular Pgp, did not significantly impact the activity of AMG 330 –a reassuring finding given the frequent expression of such transporters in human AML and their association with poor prognosis [26, 27].

In myeloid cell lines engineered to overexpress various amounts of CD33, our previous studies demonstrated that AMG 330-induced cytotoxicity was proportional to the amount of CD33 expressed on AML cells [16]. This observation suggests that low CD33 expression could be a limiting factor for AMG 330 activity in the clinical use of this BiTE antibody construct as many human AML cells only display low levels of CD33. In fact, although CD33 is found on at least a subset of blasts in nearly all AML patients [15, 28], surface levels show considerable inter-patient variability (>2-log fold) [15, 28, 29]. Overall, CD33 expression is relatively limited with an average of ~10^4^ molecules/AML blast [29, 30] and is typically even lower in immature AML cell subsets [15, 31]. Compared to our previous studies with engineered cell lines, demonstration of a relationship between CD33 expression levels and AMG 330-induced cytotoxicity was more difficult in primary AML specimens. There was no association between these 2 parameters in the absence of healthy donor T-cells. In the presence of healthy donor T-cells, only at high E:T cell ratio (3:1) and higher AMG 330 doses (250 and 500 pg/mL) could we demonstrate a statistically significant correlation between drug-induced cytotoxicity and CD33 expression levels on myeloid blasts. This observation may be partly due to the fact that high cytotoxic activity was observed in several specimens with minimal CD33 expression, even in the sole presence of autologous T-cells. Furthermore, CD33 expression levels are not randomly distributed across the spectrum of human AML but, rather, correlated with disease characteristics. For example, high levels of CD33 are associated with *NPM1* as well as high allelic *FLT3*/ITD mutations, while expression is generally low with core-binding factor translocations [15, 28, 32]. Thus, it is interesting to speculate that other factors that are associated with CD33 expression levels on primary AML cells impact AMG 330-induced cytotoxicity, thereby masking a relationship between target antigen expression and BiTE antibody construct efficacy.

The emerging clinical data with blinatumomab in ALL suggest that BiTE antibody constructs may be largely non-cross resistant to commonly used chemotherapeutics (and possibly allotransplantation) as they can be effective in otherwise treatment-refractory patients [23–25]. Consistent with this notion, AMG 330 was broadly active in our set of AML specimens across the entire cytogenetic/molecular disease spectrum not only in specimens from previously untreated AML patients but also in those from patients who failed conventional chemotherapeutic treatments and regardless of Pgp activity. These findings differ from studies with GO, for which preclinical studies have indicated reduced cytotoxic effects in specimens with adverse-risk cytogenetics or Pgp activity [33]. Attempting to translate our findings to the potential clinical development of AMG 330, these data would suggest the testing of AMG 330 across all cytogenetic/molecular subsets of AML rather than its exploration upfront in a small, defined patient subset. Nevertheless, there was significantly lower activity of AMG 330 in relapsed/refractory AML specimens relative to newly diagnosed AML specimens despite similar expression levels of CD33 in these 2 specimen subsets in experiments in which healthy donor T-cells were added. This observation identifies disease stage as rationale risk-stratification parameter for the clinical testing of AMG 330. More fundamentally, this observation also hints at the presence of one or more CD33-independent, relative resistance mechanisms in patients with prior exposure to conventional chemotherapy agents and suggests the possibility of some albeit limited cross-resistance. The nature of these resistance mechanisms, which once identified could then serve as biomarkers for risk-stratification and/or response prediction, are currently unknown. Until such mechanisms are fully understood, this observation indicates the need for careful patient stratification according to disease stage in the analysis of clinical data with AMG 330.

Although our findings are significantly limited by the small number of specimens in this patient subset, we also observed higher activity of AMG 330 in the 5 specimens from patients with favorable cytogenetic or molecular disease features as compared to the other specimens in experiments in which healthy donor T-cells were added. Unfortunately, while cytogenetic profiling was complete, molecular profiling was incomplete in many of the studied specimens and some samples with favorable-risk features (e.g. NPM1 mutation without FLT3/ITD or CEBPA double mutation) may not have been correctly categorized, potentially limiting this analysis. Thus, although sample misclassification would likely lead to a reduction of the difference between favorable-risk and other specimens, further studies with larger sets of specimens may be required to confirm our findings.

In summary, our data show that AMG 330 causes potent dose- and T-cell-dependent cytolysis of primary human AML cells *in vitro* even in cases of very low CD33 expression on myeloid blasts. Lower activity of AMG 330 was observed in relapsed/refractory AML specimens (relative to newly diagnosed AML specimens) and perhaps intermediate- or adverse-risk disease specimens (relative to favorable-risk specimens) suggesting the potential presence of yet undefined, CD33-independent, relative resistance mechanisms in defined patient subsets. Further laboratory studies will be required to identify the nature of these resistance mechanisms. Nonetheless, our data suggest that AMG 330 is broadly active against human AML, and support our conclusion that it should be clinically explored in patients with AML and, by extrapolation, perhaps other CD33^+^ hematopoietic malignancies.

## Supporting Information

S1 Fig Analysis strategy. (PDF)

S2 Fig Selection of primary AML specimens for study. (PDF)

S3 Fig Relationship between age and AMG 330-induced cytotoxicity. (PDF)

S4 Fig Relationship between Pgp activity and AMG 330-induced cytotoxicity. (PDF)

S5 Fig AMG 330-induced cytotoxicity without addition of healthy donor T-cells, restricted dataset (n = 25). (PDF)

S6 Fig AMG 330-induced cytotoxicity in the presence of healthy donor T-cells, restricted dataset (n = 25). (PDF)

S1 Table Additional Patient Characteristics, Stratified by Cytogenetic/Molecular Risk. (PDF)

S2 Table Patient Characteristics, Restricted Dataset. (PDF)
