# Supplementary material for: The Broad Anti-AML Activity of the CD33/CD3 BiTE Antibody Construct, AMG 330, Is Impacted by Disease Stage and Risk
Source: PLoS One. 2015 Aug 25;10(8):e0135945. doi: 10.1371/journal.pone.0135945 (PMC4549148; doi:10.1371/journal.pone.0135945)
Supplement: S1 Fig — (PDF) [file pone.0135945.s001.pdf]

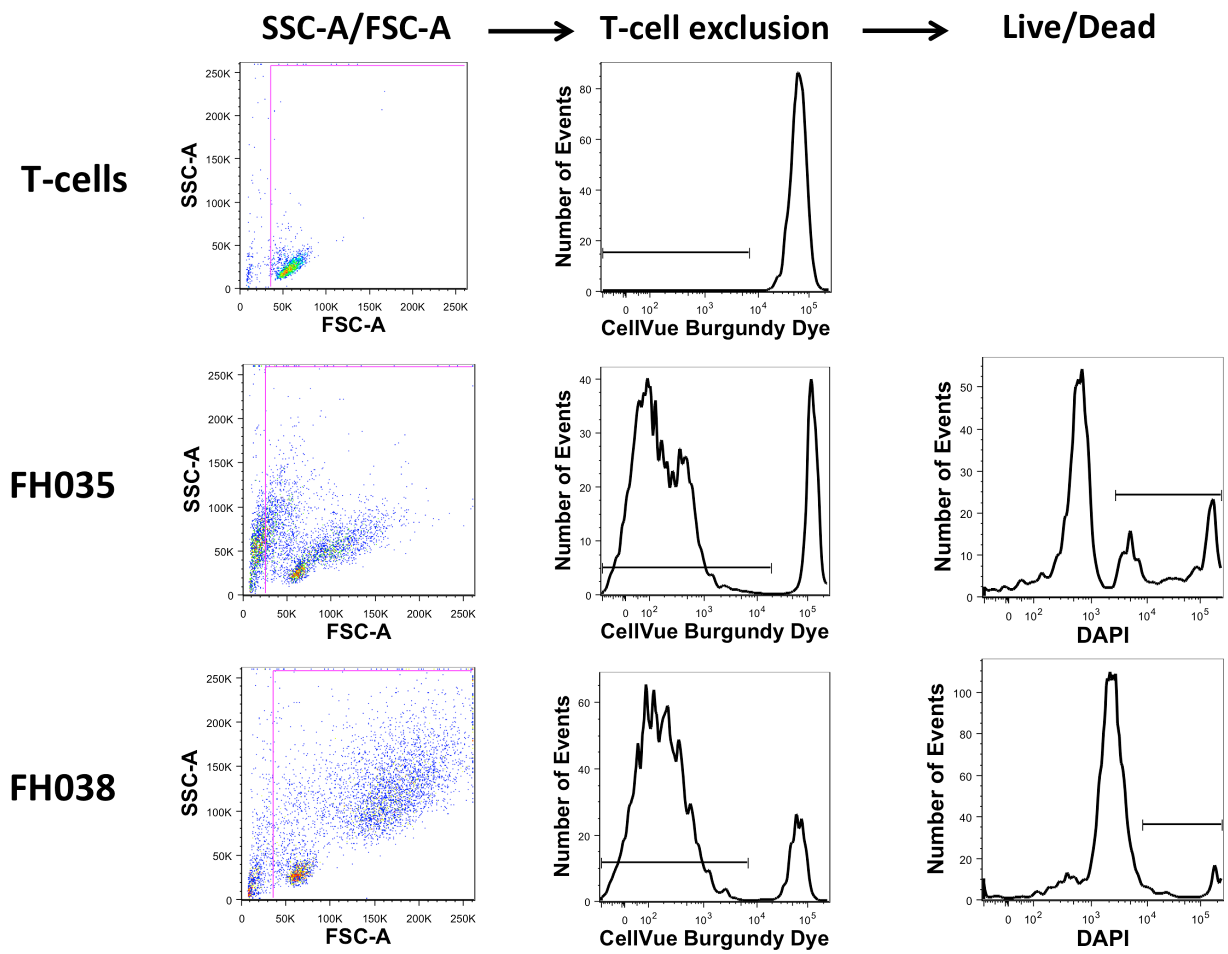

S1 Fig. Analysis strategy. Scatter and histogram plots from one healthy donor T-cell aliquot and 2 representative primary AML specimens illustrating the strategy pursued to determine AMG 330-induced cytotoxicity. FSC, forward scatter; SSC, side scatter.
