# Supplementary material for: The Broad Anti-AML Activity of the CD33/CD3 BiTE Antibody Construct, AMG 330, Is Impacted by Disease Stage and Risk
Source: PLoS One. 2015 Aug 25;10(8):e0135945. doi: 10.1371/journal.pone.0135945 (PMC4549148; doi:10.1371/journal.pone.0135945)
Supplement: S2 Fig — (PDF) [file pone.0135945.s002.pdf]

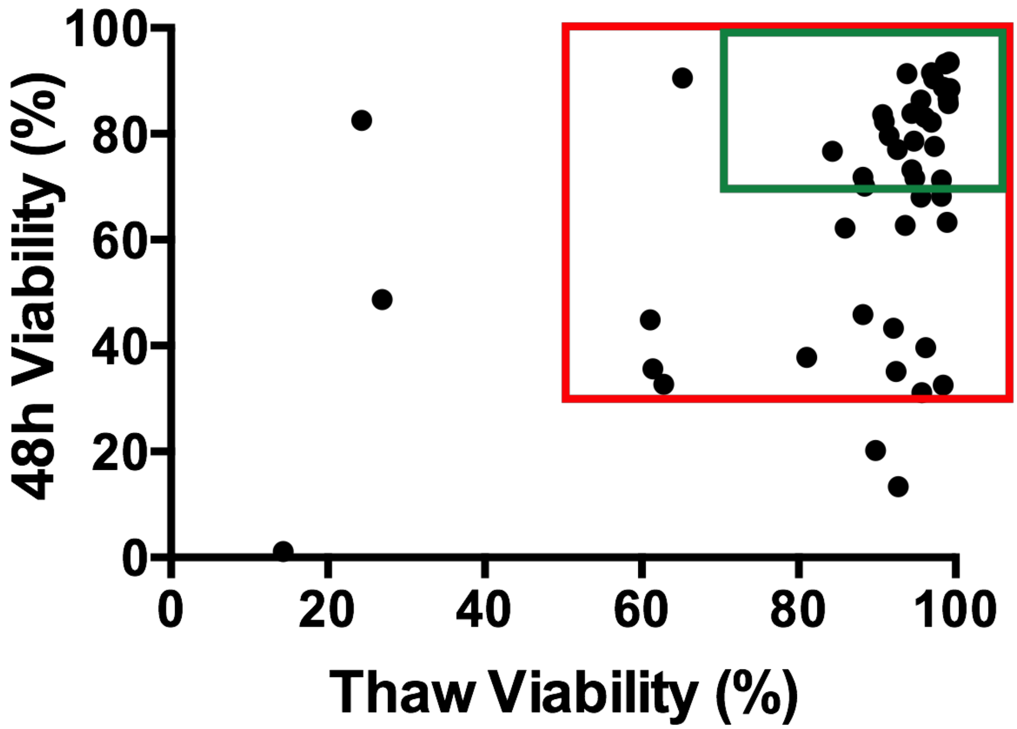

S2 Fig. Selection of primary AML specimens for study. Frozen Aliquots from a total of 49 primary human AML specimens were obtained for analysis. The percentage of AML blasts upon thaw was determined by flow cytometry based on CD45/side-scatter properties. Viability of the specimens was determined upon thaw as well after 48 hours in cytokine-containing liquid culture (without addition of AMG 330 or healthy donor T-cells) via flow cytometry using DAPI as live/dead cell marker. Results for viability upon thaw as well as after 48 hours are depicted for the 47 specimens with >40% AML blasts. **Red square:** 41 primary AML specimens that showed a viability of >50% at thaw as well as >30% after 48 hours in cytokine-containing liquid culture. **Green square:** subset of 25 primary AML specimens that showed a viability of >70% both at thaw as well as after 48 hours in cytokine-containing liquid culture.
