# Supplementary material for: The Broad Anti-AML Activity of the CD33/CD3 BiTE Antibody Construct, AMG 330, Is Impacted by Disease Stage and Risk
Source: PLoS One. 2015 Aug 25;10(8):e0135945. doi: 10.1371/journal.pone.0135945 (PMC4549148; doi:10.1371/journal.pone.0135945)
Supplement: S4 Fig — (PDF) [file pone.0135945.s004.pdf]

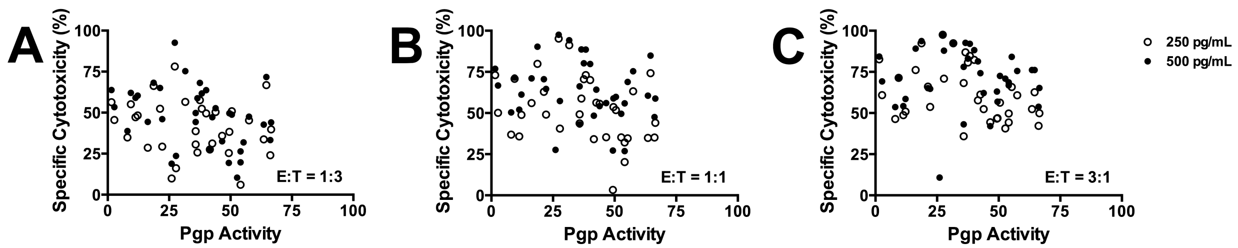

S4 Fig. Relationship between Pgp activity and AMG 330-induced cytotoxicity.

Relationship between Pgp activity and drug-induced cytotoxicity with AMG 330 at 250 pg/mL (open symbol) and 500 pg/mL (closed symbol) in the presence of T-cells from a single healthy donor at an E:T cell ratio of **(A)** 1:3, **(B)** 1:1, and **(C)** 3:1, determined by flow cytometry after 48 hours.
