# Supplementary material for: The Broad Anti-AML Activity of the CD33/CD3 BiTE Antibody Construct, AMG 330, Is Impacted by Disease Stage and Risk
Source: PLoS One. 2015 Aug 25;10(8):e0135945. doi: 10.1371/journal.pone.0135945 (PMC4549148; doi:10.1371/journal.pone.0135945)
Supplement: S5 Fig — (PDF) [file pone.0135945.s005.pdf]

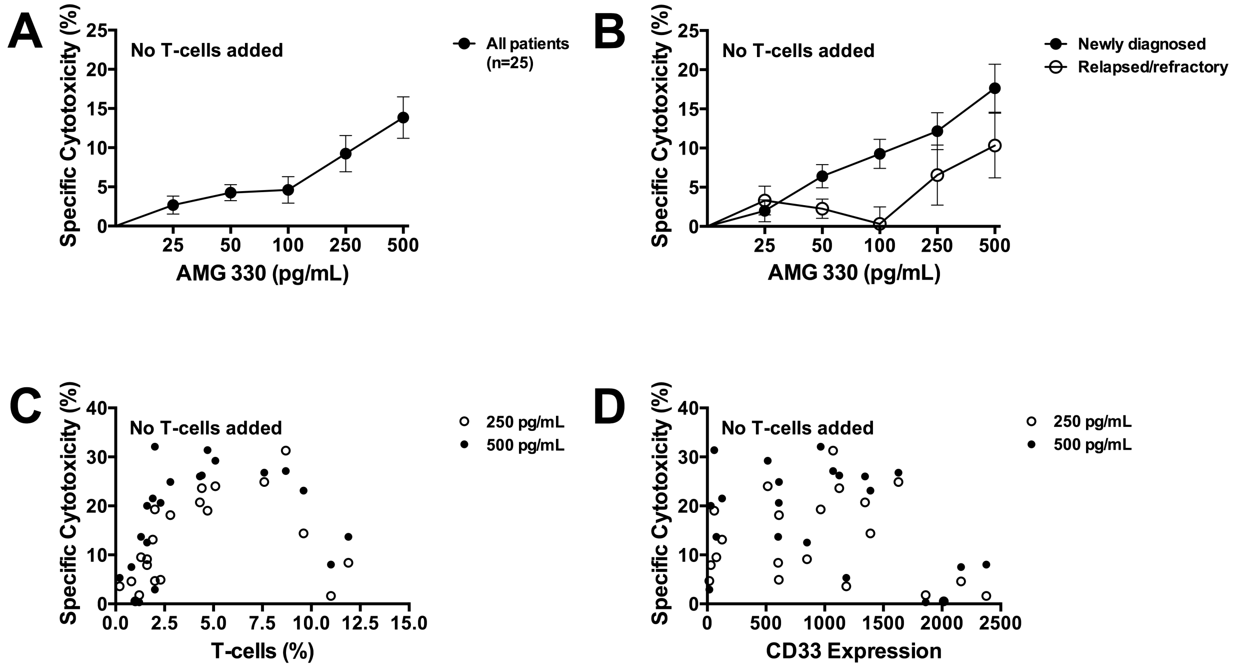

S5 Fig. AMG 330-induced cytotoxicity without addition of healthy donor T-cells, restricted dataset (n=25). (A) 48 hour AMG 330-induced cytotoxicity in the 25 primary AML specimens with highest baseline *in vitro* viability. **(B)** AMG 330-induced cytotoxicity, stratified by disease stage (i.e. newly diagnosed AML [n=12] vs. relapsed/refractory AML [n=13]). **(C)** Relationship between percentage of autologous T-cells and AMG 330-induced cytotoxicity; at 250 ng/mL: r=0.521, *P*=0.0076; at 500 ng/mL: r=0.537, *P*=0.0056. **(D)** Relationship between CD33 expression on leukemic blasts (expressed as arbitrary fluorescence intensity) and AMG 330-induced cytotoxicity; at 250 ng/mL: r=-0.239, *P*=0.25; at 500 ng/mL: r=-0.233, *P*=0.26.
