# Supplementary material for: The Broad Anti-AML Activity of the CD33/CD3 BiTE Antibody Construct, AMG 330, Is Impacted by Disease Stage and Risk
Source: PLoS One. 2015 Aug 25;10(8):e0135945. doi: 10.1371/journal.pone.0135945 (PMC4549148; doi:10.1371/journal.pone.0135945)
Supplement: S6 Fig — (PDF) [file pone.0135945.s006.pdf]

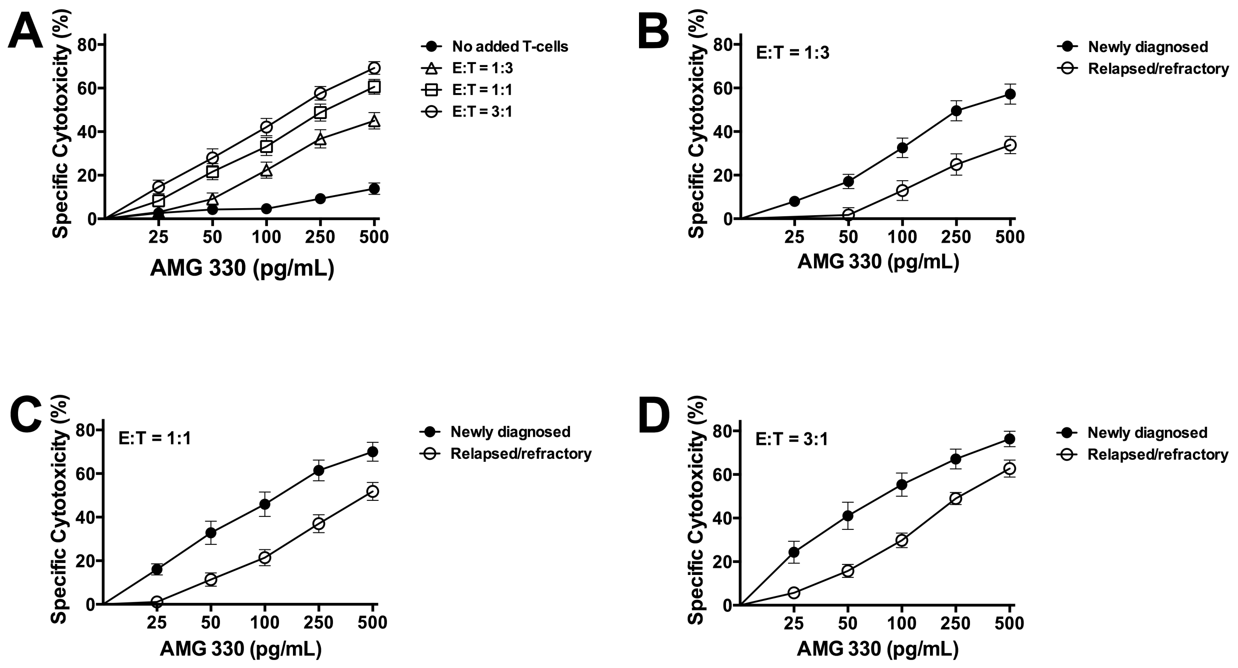

S6 Fig. AMG 330-induced cytotoxicity in the presence of healthy donor T-cells, restricted dataset (n=25). (A) 48 hour AMG 330-induced cytotoxicity in the 25 primary AML specimens with highest baseline *in vitro* viability with and without added healthy donor T-cells, as indicated. **(B, C, D)** AMG 330-induced cytotoxicity at 48 hours, stratified by disease stage (newly diagnosed AML [n=12] and relapsed/refractory AML [n=13]) in the presence of T-cells from a single healthy donor at E:T cell ratios of 1:3, 1:1, and 3:1 as indicated.
