# Supplementary material for: The Broad Anti-AML Activity of the CD33/CD3 BiTE Antibody Construct, AMG 330, Is Impacted by Disease Stage and Risk
Source: PLoS One. 2015 Aug 25;10(8):e0135945. doi: 10.1371/journal.pone.0135945 (PMC4549148; doi:10.1371/journal.pone.0135945)
Supplement: S1 Table — (PDF) [file pone.0135945.s007.pdf]

S1 Table. Additional Patient Characteristics, Stratified by Cytogenetic/Molecular Risk

	**Favorable-Risk** (n=5)	**Intermediate-Risk** (n=26)	**Adverse-Risk** (n=10)
**Median age (range), years**	61.1 (23.9-67.4)	62.9 (34.0-79.3)	71.5 (26.2-80.0)
**Specimen source**			
Bone marrow	2	9	11
Peripheral blood	3	12	9
**Median % blasts (range)**	92.3 (80.2-94.1)	86.0 (55.1-95.1)	88.5 (58.7-97.0)
**Median CD33 expression on blasts (range)**	849 (125-2,136)	1264 (59-5,356)	289 (7-3,294)
**Median % T-cells (range)**	0.1 (0-15.3)	2.8 (0.2-27.3)	2.0 (0-5.1)
**Median Ppg activity on blasts (range)**	27.3 (0-50.5)	36.5 (0-66.5)	38.0 (0-64.6)
**Median % viability at 48 hours (range)**	71.8 (68.0-90.5)	77.7 (32.7-93.5)	74.2 (31.1-83.6)
