# Supplementary material for: The Broad Anti-AML Activity of the CD33/CD3 BiTE Antibody Construct, AMG 330, Is Impacted by Disease Stage and Risk
Source: PLoS One. 2015 Aug 25;10(8):e0135945. doi: 10.1371/journal.pone.0135945 (PMC4549148; doi:10.1371/journal.pone.0135945)
Supplement: S2 Table — (PDF) [file pone.0135945.s008.pdf]

S2 Table. **Patient Characteristics, Restricted Dataset**

	**All patients** (n=25)	**Newly diagnosed AML** (n=12)	**Relapsed/refractory AML** (n=13)
**Median age (range), years**	60.4 (23.9-80.0)	65.9 (23.9-80.0)	48.5 (26.2-76.4)
**Cytogenetic/molecular risk**			
Favorable	1	1	--
Intermediate	18	8	10
CEBPA$^{double\text{-}mutant}$	1	1	--
NPM1^{pos}/*FLT3-ITD*^{neg}	1	1	--
Adverse	6	3	3
**Specimen source**			
Bone marrow	14	6	8
Peripheral blood	11	6	5
**Median % blasts (range)**	86.1 (58.4-94.1)	88.1 (58.7-94.1)	85.9 (58.4-93.4)
**Median CD33 expression on blasts (range)**	966 (18-2,377)	729 (30-2,162)	1,071 (18-2,377)
**Median % T-cells (range)**	2.1 (0.2-11.9)	2.3 (0.8-11.9)	2.1 (0.2-11.0)
**Median Pgp activity on blasts (range)**	43.3 (0-66.5)	41.6 (2.7-64.6)	49.3 (0-66.5)
**Median % viability at 48 hours (range)**	83.1 (70.1-93.5)	78.3 (70.1-93.2)	83.9 (71.6-93.5)
